# Supplementary material for: WUSCHEL acts as an auxin response rheostat to maintain apical stem cells in Arabidopsis
Source: Nat Commun. 2019 Nov 8;10:5093. doi: 10.1038/s41467-019-13074-9 (PMC6841675; doi:10.1038/s41467-019-13074-9)
Supplement: Supplementary file 1 — Supplementary Information [file 41467_2019_13074_MOESM1_ESM.pdf]

SUPPLEMENTARY FIGURES AND TABLES FOR

WUSCHEL acts as an auxin response rheostat to maintain apical stem cells in *Arabidopsis*

Yanfei Ma[1§], Andrej Miotk[1§], Zoran Šutiković[1§], Olga Ermakova[1], Christian Wenzl[1], Anna Medzihradszky[1], Christophe Gaillochet[1], Joachim Forner[1], Gözde Utan[1], Klaus Brackmann[2], Carlos S. Galvan-Ampudia[3], Teva Vernoux[3], Thomas Greb[4] & Jan U. Lohmann[1*]

[1]Department of Stem Cell Biology, Centre for Organismal Studies, Heidelberg University; D-69120 Heidelberg, Germany

[2]Gregor Mendel Institute (GMI), Austrian Academy of Sciences, Vienna Biocenter (VBC), Dr. Bohr-Gasse 3, 1030 Vienna, Austria

[3]Laboratoire Reproduction et Développement des Plantes, Univ Lyon, ENS de Lyon, UCB Lyon 1, CNRS, INRA, F-69342 Lyon, France

[4]Department of Developmental Physiology, Centre for Organismal Studies, Heidelberg University; D-69120 Heidelberg, Germany

[§] these authors contributed equally

*corresponding author

Mailing address of corresponding author:

Jan U. Lohmann
Department of Stem Cell Biology
Heidelberg University
Im Neuenheimer Feld 230
D-69120 Heidelberg
Germany

PH: +49 6221 546269
FX: +49 6221 546424
EM: jlohmann@meristemania.org

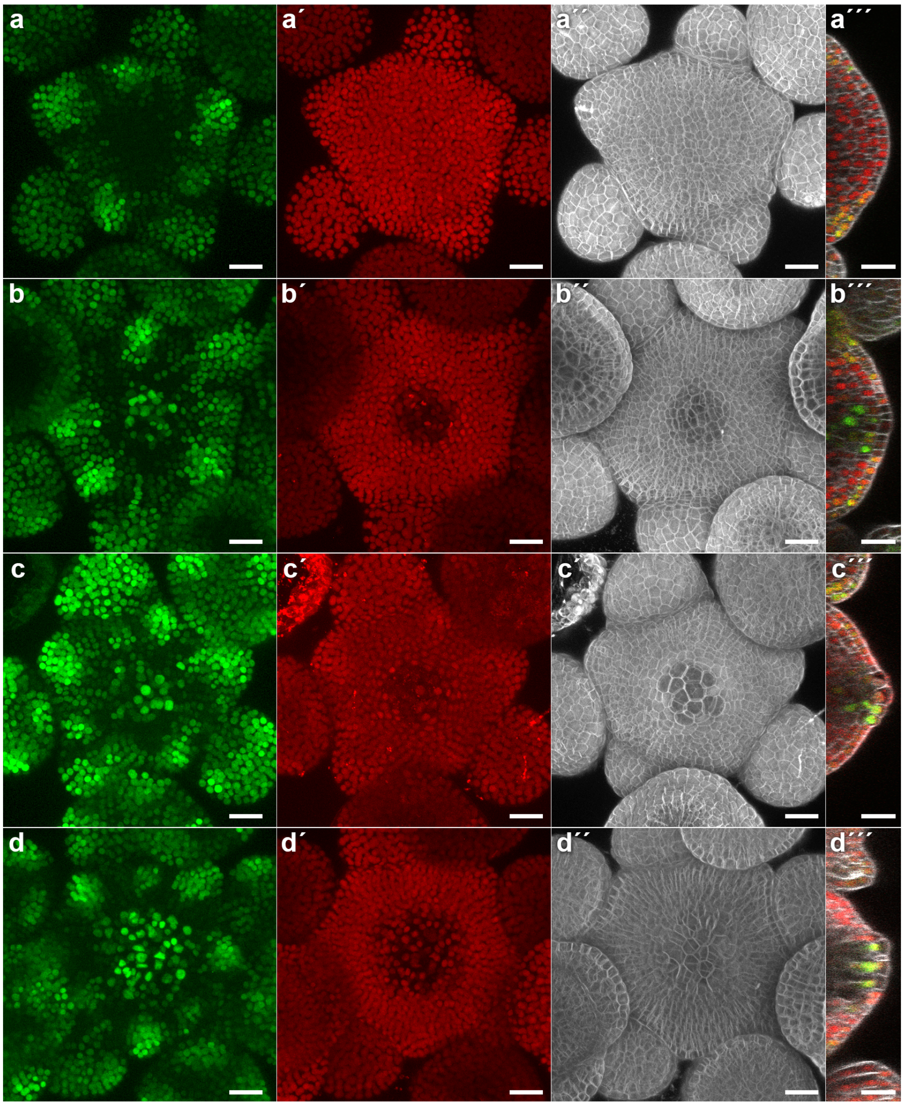

Supplementary Fig. 1: Activation of DR5v2 and differentiation after induced stem cell loss.

Representative SAMs of the imaged cohorts quantified in Fig. 2f and Supplementary Fig 2. A) 0h after induction B) 36h after induction C) 72h after induction D) 120h after induction. Left panels show *pDR5v2:3xVENUS-NLS* signal, middle panels show *pRPS5a:NLS-tdTomato* and right panels show DAPI stained cell walls. Small panels on the far-right show Z-projections of all channels through the center of the SAM. Scale bars 20 μm.

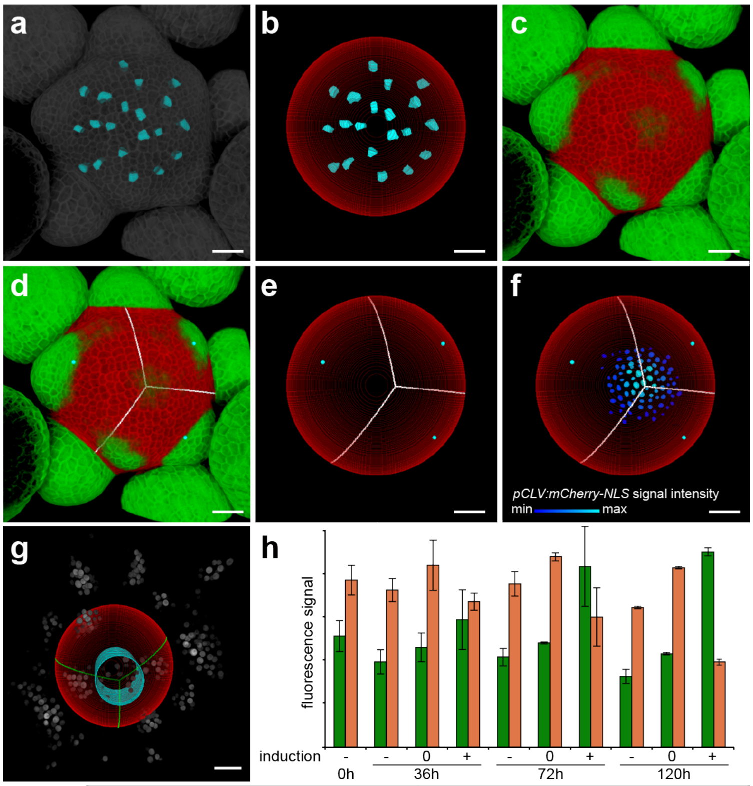

i

	induced			untreated		
	pRPS5A +	pRPS5A -	DR5v2 +	pRPS5A +	pRPS5A -	DR5v2 +
0h	-	-	-	7	0	0
36h	3	**4**	**4**	7	0	0
72h	2	**5**	**5**	7	0	0
120h	2	**2**	**2**	4	0	0

Supplementary Figure 2: Computational strategy to identify stem cells and DR5v2 quantification.

a) In a first step, cells across the L1 of the SAM are segmented. **b)** Based on the position of segmented cells, a perfect sphere is fitted to the SAM. **c)** The sphere is applied to the SAM and organ primordia are identified by emergence through the sphere. **d, e)** Equidistant points between the primordia are calculated and used to triangulate the center of the SAM. **f)** The triangulated center was benchmarked against SAMs harboring *pCLV3* reporter labelled stem cells (n=9). The triangulation invariantly identified one of the most central *pCLV3* positive cells. See also Methods. **g)** For signal quantification in the stem cell domain, a cylinder with radius r_{cyl} (= 1/3 * r_{sphere}) mimicking the average size of the *CLV3* domain was placed into the computationally identified center of the SAM and fluorescence intensities were quantified within this narrowly defined subdomain. DR5v2-NLS signals are shown in grey, SAM sphere derived from segmentation in red, triangulation lines in green and quantification cylinder in cyan. **h)** Quantification of fluorescent signals from all SAMs of the stem cell

loss experiment described in Fig. 2 and Supplementary Fig. 1. Total fluorescence signal intensities for *pDR5v2:3xVENUS-NLS* and *pRPS5a:NLS-tdTomato* for the inner region (I_{cyl}) and for the peripheral region (I_{sphere}) were extracted from respective image volumes. I_{cyl} was averaged over all plants for each time-point and condition and normalized to the overall signal (I_{cyl} + I_{sphere}). Green bars: DR5v2:3xVENUS-NLS signal, orange bars: pRPS5a:NLS-tdTomato signal. - : mock treated, 0: ethanol induced, but no observable stem cell loss, + : ethanol induced and stem cell loss. **i)** Quantification of DR5v2 response to induction of iCalSm at the per plant level. Number of plants scored for loss of *RPS5a* promoter activity from stem cells and DR5v2 expression are shown. Stem cell loss and associated DR5v2 activation exclusively occurred in induced plants. All plants with stem cell loss as shown by reduced *pRPS5a* activity expressed DR5v2. *pRPS5a* + denotes plants with uncompromised *pRPS5a* promoter activity in stem cells. *pRPS5a* - denotes plants with reduced *pRPS5a* promoter activity in stem cells. DR5v2 + denotes plants with DR5v2 activity in stem cells. Scale bars 20 μm.

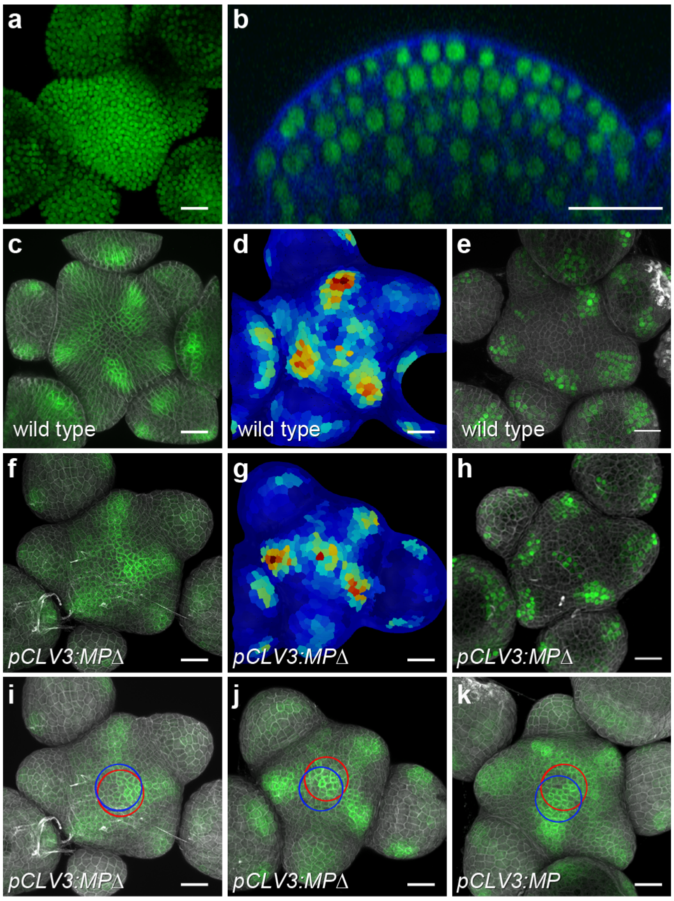

Supplementary Figure 3: Activity of the *pHMG* promoter, behavior of nuclear and ER localized DR5v2 reporters and auxin signaling output in wild type and *pCLV3:MPΔ* lines.

a, b) Transgenic line carrying 1347 bp upstream of the At1g76110 locus fused to the *GFP-NLS* coding sequence. **a)** GFP channel in top view. **b)** Side view though a representative SAM showing DAPI and GFP channel. **c)** *pDR5v2:ER-EYFP-HDEL* in wild type. **d)** Per cell quantification of an independent *pDR5v2:ER-EYFP-HDEL* wild-type SAM. **e)** *pDR5v2:3xVENUS-NLS* in wild type. **f-k)** Auxin signaling output was present in the center of *pCLV3:MP* and *pCLV3:MPΔ* lines, indicated by two independent reporters *pDR5v2:ER-EYFP-HDEL* (6 out of 8 independent T1 plants) (F) and *pDR5v2:3xVENUS-NLS* (6 out of 7 independent T1 plants) (H). **g)** Per cell quantification of *pDR5v2:ER-EYFP-HDEL* in an independent *pCLV3:MPΔ* SAM. DR5v2 activity was not observed in the center of wild-type SAMs grown in the same experiments. **i-k)** Computationally derived central zone in L1 (red) and L3 (blue) are superimposed to SAMs of *pDR5v2:ER-EYFP-HDEL* carrying *pCLV3:MPΔ* (I, J) and *pCLV3:MP* (K). DR5v2 signal clearly coincides with central zone. Scale bars 20 μm.

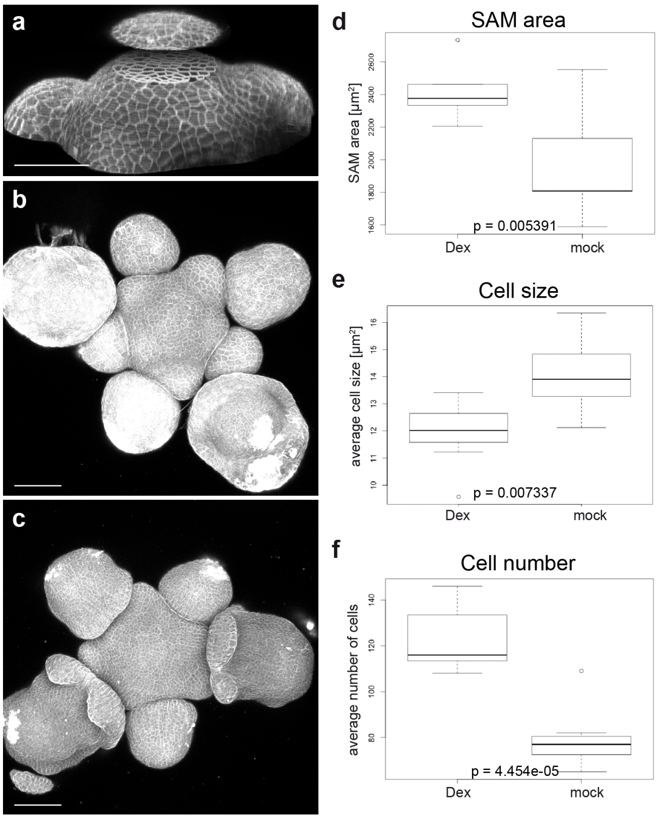

Supplementary Figure 4: Morphological effects of WUS-GR induction.

a) Visualization of meristem morphology analysis strategy. Meristem size, cell count and average cell size were measured at a constant relative position defined by the image plane in which the L1 to L2 transition became visible. **b)** Mock treated and **c)** DEX induced *pUBI:mCherry-GR-linker-WUS* SAMs four days after local application. Following WUS induction, SAM size increased **(d)**, cell size decreased **(e)** and cell number strongly increased **(f)** n= 7 and 8 meristems, respectively. p-values derived by t-test. Scale bars 50 *µ*m.

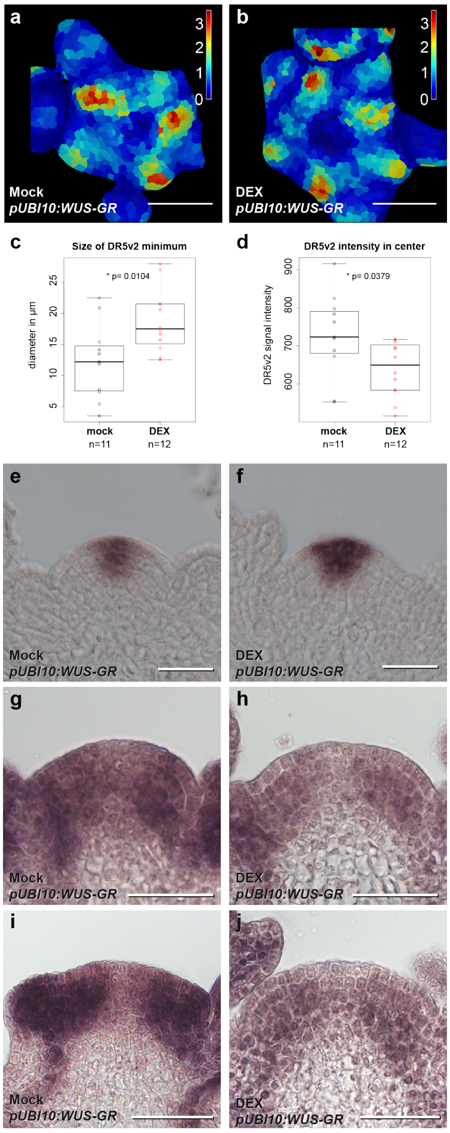

Supplementary Figure 5: SAM specific molecular responses to ectopic WUS induction in *pUBI10:WUS-GR lines*.

24 hours after induction of ectopic WUS-GR activity, DR5v2 signal in the central zone was suppressed and *CLV3* mRNA expression was enhanced. Representative in situ quantifications of DR5v2 signal after mock **(a)** and DEX **(b)** treatments. **c)** Quantification of the size of the central DR5v2 minimum. **d)** Quantification of the average DR5v2 signal intensity in the central zone. p-values derived by t-test. **e)** *CLV3* mRNA expression after 24 hours of mock treatment. **f)** *CLV3* mRNA expression after 24 hours of DEX treatment. **g)** *TIR1* mRNA expression after 24 hours of mock treatment. **h)** *TIR1* mRNA expression after 24 hours of DEX treatment. **i)** *MP* mRNA expression after 24 hours of mock treatment. **j)** *MP* mRNA expression after 24 hours of DEX treatment. SAMs of both treatment types were hybridized on the same microscopic slide and imaged under identical settings. Scale bars 50 μm.

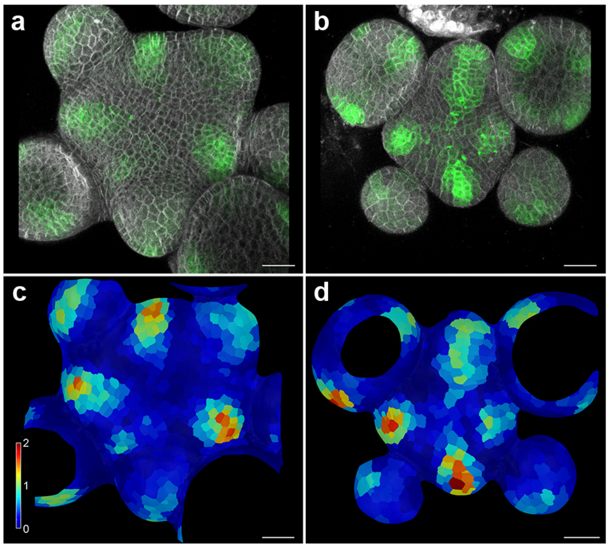

Supplementary Figure 6: SAMs of *wus-7* plants show auxin signaling output in the stem cell domain.

a) Representative image of *pDR5v2:ER-eYFP-HDEL* signal in the SAM of L*er* wild-type plants. Only 16% of plants showed DR5v2 activity in the center of the SAM (n=38). **b)** Representative image of *pDR5v2:ER-eYFP-HDEL* signal in a *wus-7* SAM before termination. 61% of *wus-7* plants showed DR5v2 activity in the center of the SAM (n=13). Per cell quantification of DR5v2 signal in wild type (**c**) and *wus-7* (**d**). Scale bars 20 μm.

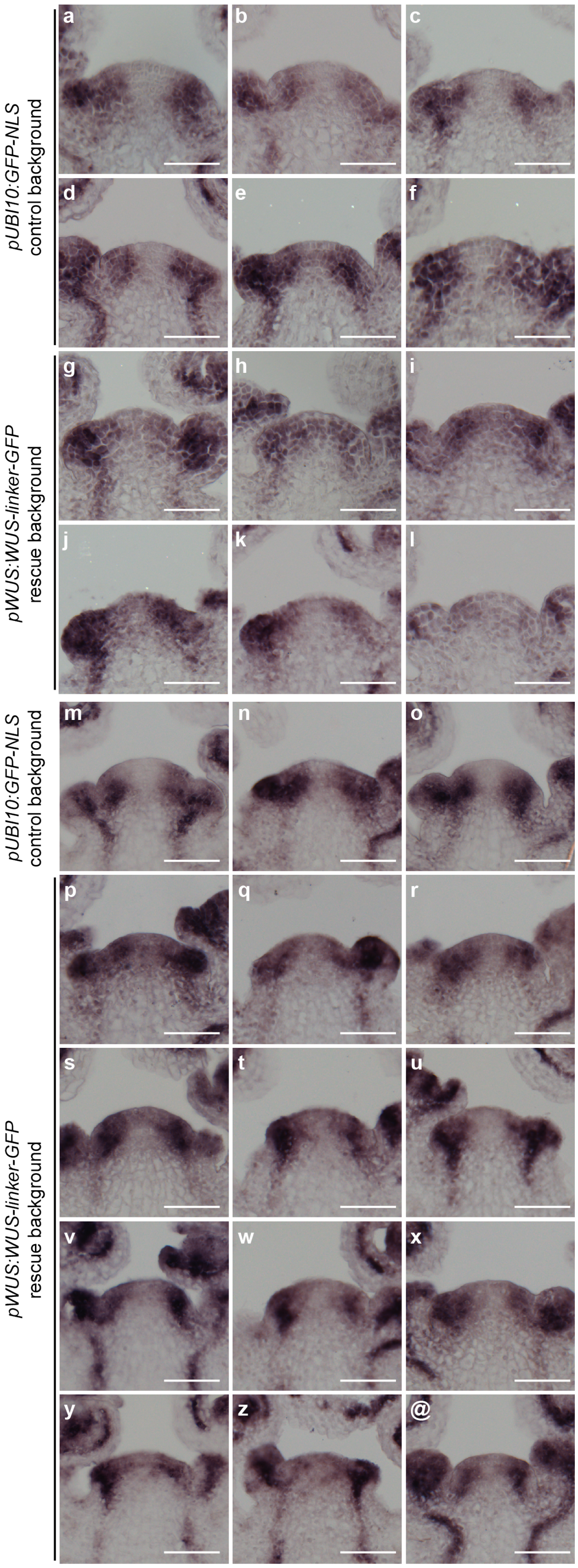

Supplementary Figure 7: *MP* mRNA expression after induced WUS loss of function in two independent experiments.

a-l) Experiment I. a-f) In situ detection of *MP* mRNA in *pUBQ10:GFP-NLS* control plants carrying *pCLV3:AlcR/AlcA:NSlmb-vhhGFP4* after 24h of ethanol treatment. **g-l)** In situ detection of *MP* mRNA in stable *pWUS:WUS-linker-GFP wus* rescue plants carrying *pCLV3:AlcR/AlcA:NSlmb-vhhGFP4* after 24h of ethanol treatment.

m-@) Experiment II. m-o) In situ detection of *MP* mRNA in *pUBQ10:GFP-NLS* control plants carrying *pCLV3:AlcR/AlcA:NSlmb-vhhGFP4* after 24h of ethanol treatment. **p-@)** In situ detection of *MP* mRNA in stable *pWUS:WUS-linker-GFP wus* rescue plants carrying *pCLV3:AlcR/AlcA:NSlmb-vhhGFP4* after 24h of ethanol treatment.

SAMs of both genotypes were hybridized in sets of two independent experiments and imaged under identical settings. Unadjusted images are shown. Scale bars 50 μm.

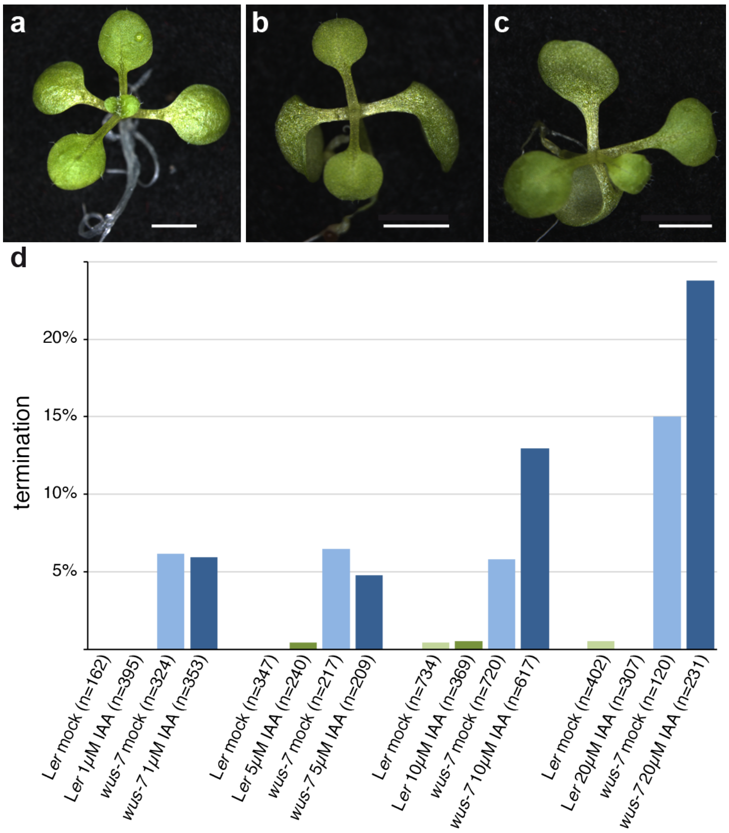

Supplementary Figure 8: Seedling phenotypes eleven days after germination on auxin supplemented plates and SAM termination frequencies on increasing auxin concentrations.

Phenotypes of seedlings grown on plates containing 10 *µ*m IAA ranged from **a)** unaffected, to **b)** arrested at four leaves stage, or **c)** arrested at five leaves stage with a terminal leaf. **d)** Quantification of terminated seedlings grown on plates containing various concentrations of auxin. Wild-type L*er* and *wus-7* seedlings were tested on 1-20 *µ*M IAA, termination in wus-7 starts to occur on 10*µ*M IAA, wild type is resistant to even 20 *µ*M IAA. Scale bars 1 mm.

	GO ID	Term	Annotated genes	Significant genes	Expected	p-Value
**1**	GO:0010200	response to chitin	393	145	55.45	2.80E-30
**2**	GO:0009611	response to wounding	313	109	44.16	1.00E-20
**3**	GO:0010363	regulation of plant-type hypersensitive response	336	111	47.41	4.60E-19
**4**	GO:0006612	protein targeting to membrane	340	111	47.97	1.30E-18
**5**	GO:0009414	response to water deprivation	374	130	52.77	5.70E-18
**6**	GO:0009867	jasmonic acid mediated signaling pathway	256	89	36.12	1.20E-15
**7**	GO:0009733	response to auxin	354	107	49.95	2.30E-15
**8**	GO:0002679	respiratory burst involved in defense response	114	50	16.09	1.10E-14
**9**	GO:0009737	response to abscisic acid	548	174	77.32	1.10E-14
**10**	GO:0009738	abscisic acid-activated signaling pathway	232	78	32.74	1.10E-12
**11**	GO:0009651	response to salt stress	704	187	99.33	2.60E-12
**12**	GO:0009695	jasmonic acid biosynthetic process	125	49	17.64	3.40E-12
**13**	GO:0006857	oligopeptide transport	97	41	13.69	1.10E-11
**14**	GO:0050832	defense response to fungus	303	84	42.75	3.30E-10
**15**	GO:0009862	systemic acquired resistance. salicylic acid mediated signaling pathway	222	66	31.32	1.20E-09
**16**	GO:0042538	hyperosmotic salinity response	152	50	21.45	2.90E-09
**17**	GO:0009612	response to mechanical stimulus	59	27	8.32	4.50E-09
**18**	GO:0042742	defense response to bacterium	344	93	48.54	4.90E-09
**19**	GO:0009684	indoleacetic acid biosynthetic process	94	36	13.26	5.20E-09
**20**	GO:0006569	tryptophan catabolic process	67	29	9.45	6.00E-09
**21**	GO:0009723	response to ethylene	325	101	45.86	1.20E-08
**22**	GO:0009753	response to jasmonic acid	427	141	60.25	1.20E-08
**23**	GO:0009873	ethylene-activated signaling pathway	118	41	16.65	1.30E-08
**24**	GO:0009620	response to fungus	440	132	62.08	2.50E-08
**25**	GO:0000165	MAPK cascade	197	57	27.8	4.50E-08
**26**	GO:0009963	positive regulation of flavonoid biosynthetic process	93	34	13.12	5.30E-08
**27**	GO:0006355	regulation of transcription. DNA-templated	1588	296	224.07	7.10E-08
**28**	GO:0043069	negative regulation of programmed cell death	158	48	22.29	1.00E-07
**29**	GO:0009739	response to gibberellin	143	49	20.18	1.10E-07
**30**	GO:0031348	negative regulation of defense response	246	65	34.71	2.30E-07
**31**	GO:0009409	response to cold	539	118	76.05	4.20E-07
**32**	GO:0009750	response to fructose	127	39	17.92	1.10E-06
**33**	GO:0030968	endoplasmic reticulum unfolded protein response	171	48	24.13	1.30E-06
**34**	GO:0009693	ethylene biosynthetic process	110	35	15.52	1.60E-06
**35**	GO:0009805	coumarin biosynthetic process	51	21	7.2	2.00E-06
**36**	GO:0010310	regulation of hydrogen peroxide metabolic process	159	45	22.43	2.20E-06
**37**	GO:0030003	cellular cation homeostasis	146	42	20.6	3.00E-06
**38**	GO:0007623	circadian rhythm	156	44	22.01	3.20E-06
**39**	GO:0006833	water transport	118	36	16.65	3.40E-06
**40**	GO:0009741	response to brassinosteroid	102	37	14.39	3.60E-06
**41**	GO:0080167	response to karrikin	114	35	16.09	4.00E-06
**42**	GO:0002237	response to molecule of bacterial origin	97	31	13.69	5.60E-06
**43**	GO:0006979	response to oxidative stress	407	90	57.43	6.50E-06
**44**	GO:0006813	potassium ion transport	35	16	4.94	6.60E-06
**45**	GO:0046777	protein autophosphorylation	131	37	18.48	1.80E-05
**46**	GO:0006598	polyamine catabolic process	34	15	4.8	2.20E-05
**47**	GO:0035556	intracellular signal transduction	446	133	62.93	2.30E-05
**48**	GO:0009269	response to desiccation	31	14	4.37	3.00E-05
**49**	GO:0031347	regulation of defense response	485	146	68.43	3.00E-05
**50**	GO:0009825	multidimensional cell growth	96	29	13.55	3.70E-05
**51**	GO:0009697	salicylic acid biosynthetic process	181	46	25.54	3.70E-05
**52**	GO:0019344	cysteine biosynthetic process	181	46	25.54	3.70E-05
**53**	GO:0006970	response to osmotic stress	749	207	105.68	4.10E-05
**54**	GO:0070838	divalent metal ion transport	184	53	25.96	6.90E-05
**55**	GO:0009627	systemic acquired resistance	395	109	55.73	7.70E-05
**56**	GO:0006949	syncytium formation	19	10	2.68	8.30E-05
**57**	GO:0042398	cellular modified amino acid biosynthetic process	50	18	7.06	9.10E-05
**58**	GO:0009751	response to salicylic acid	423	122	59.69	9.80E-05
**59**	GO:0042631	cellular response to water deprivation	59	20	8.32	1.00E-04
**60**	GO:0009965	leaf morphogenesis	186	49	26.24	1.10E-04
**61**	GO:0010583	response to cyclopentenone	132	35	18.63	1.20E-04
**62**	GO:0001666	response to hypoxia	74	23	10.44	1.40E-04
**63**	GO:0007030	Golgi organization	160	40	22.58	1.70E-04
**64**	GO:0016126	sterol biosynthetic process	150	38	21.17	1.80E-04
**65**	GO:0019748	secondary metabolic process	527	133	74.36	2.20E-04
**66**	GO:0006468	protein phosphorylation	620	157	87.48	2.40E-04
**67**	GO:0006995	cellular response to nitrogen starvation	21	10	2.96	2.40E-04
**68**	GO:0009863	salicylic acid mediated signaling pathway	315	92	44.45	2.80E-04
**69**	GO:0009407	toxin catabolic process	180	43	25.4	2.90E-04
**70**	GO:0009595	detection of biotic stimulus	92	26	12.98	3.00E-04

	GO ID	Term	Annotated genes	Significant genes	Expected	p-Value
**71**	GO:0046686	response to cadmium ion	415	84	58.56	3.30E-04
**72**	GO:0006816	calcium ion transport	108	29	15.24	3.60E-04
**73**	GO:0042335	cuticle development	42	15	5.93	3.80E-04
**74**	GO:0009617	response to bacterium	499	140	70.41	4.00E-04
**75**	GO:0010264	myo-inositol hexakisphosphate biosynthetic process	51	17	7.2	4.10E-04
**76**	GO:0010119	regulation of stomatal movement	47	16	6.63	4.60E-04
**77**	GO:0043900	regulation of multi-organism process	115	30	16.23	4.90E-04
**78**	GO:0010017	red or far-red light signaling pathway	39	14	5.5	5.60E-04
**79**	GO:0010260	animal organ senescence	27	11	3.81	6.30E-04
**80**	GO:0009740	gibberellic acid mediated signaling pathway	72	21	10.16	7.00E-04
**81**	GO:0007169	transmembrane receptor protein tyrosine kinase signaling pathway	113	29	15.94	8.00E-04
**82**	GO:0015824	proline transport	68	20	9.59	8.30E-04
**83**	GO:0010227	floral organ abscission	32	12	4.52	8.80E-04
**84**	GO:0052541	plant-type cell wall cellulose metabolic process	24	10	3.39	9.00E-04
**85**	GO:0010158	abaxial cell fate specification	7	5	0.99	9.10E-04
**86**	GO:0009742	brassinosteroid mediated signaling pathway	37	13	5.22	1.10E-03
**87**	GO:0048767	root hair elongation	164	38	23.14	1.17E-03
**88**	GO:0010118	stomatal movement	86	32	12.13	1.68E-03
**89**	GO:0009694	jasmonic acid metabolic process	147	58	20.74	1.71E-03
**90**	GO:0033500	carbohydrate homeostasis	12	8	1.69	1.74E-03
**91**	GO:0007231	osmosensory signaling pathway	5	4	0.71	1.75E-03
**92**	GO:2000022	regulation of jasmonic acid mediated signaling pathway	5	4	0.71	1.75E-03
**93**	GO:0010037	response to carbon dioxide	5	4	0.71	1.75E-03
**94**	GO:0009624	response to nematode	72	20	10.16	1.80E-03
**95**	GO:0006766	vitamin metabolic process	77	21	10.86	1.80E-03
**96**	GO:0006865	amino acid transport	228	61	32.17	2.09E-03
**97**	GO:0000038	very long-chain fatty acid metabolic process	44	14	6.21	2.14E-03
**98**	GO:0046885	regulation of hormone biosynthetic process	8	5	1.13	2.15E-03
**99**	GO:0050801	ion homeostasis	205	59	28.93	2.26E-03
**100**	GO:0052546	cell wall pectin metabolic process	40	13	5.64	2.47E-03

Supplementary Table 1: GO category enrichment analysis of direct WUS targets. Top 100 enriched categories are shown. As a background set for calculating enrichment, we used genes in open chromatin[28].

		ChIP-seq					RNA-seq	
**AGI**	**Gene Name**	**peaks**	Ac	deAc	Me	deMe	**Log2FC**	**p adj.**
**AT1G59750**	ARF1	0	-	+	-	-	-0.09	4.26E-01
**AT5G62000**	ARF2	1	+	-	-	-	-0.14	7.01E-02
**AT2G33860**	ARF3	1	+	-	-	-	0.78	2.11E-06
**AT5G60450**	ARF4	1	-	+	-	-	-1.26	4.76E-18
**AT1G19850**	ARF5	1	-	-	-	-	0.86	1.25E-04
**AT1G30330**	ARF6	5	+	+	-	-	0.29	1.40E-02
**AT5G20730**	ARF7	0	+	-	-	-	0.17	2.46E-01
**AT5G37020**	ARF8	1	-	-	-	-	-1.58	8.52E-20
**AT4G23980**	ARF9	1	-	-	-	-	1.15	5.92E-25
**AT2G28350**	ARF10	2	+	-	-	-	0.85	9.49E-04
**AT2G46530**	ARF11	1	-	-	-	+	0.93	4.27E-07
**AT1G34310***	ARF12	0	-	-	-	-	0	1
**AT1G34170***	ARF13	0	-	-	-	-	0	1
**AT1G35540***	ARF14	0	-	-	-	-	0	1
**AT1G35520***	ARF15	0	-	-	-	-	0	1
**AT4G30080**	ARF16	0	+	-	-	-	0.14	6.29E-01
**AT1G77850**	ARF17	0	+	-	-	-	0.87	4.19E-05
**AT3G61830***	ARF18	0	+	+	-	-	0.99	7.48E-15
**AT1G19220**	ARF19	0	+	-	-	-	1.12	2.96E-09
**AT1G35240***	ARF20	0	-	-	-	-	0	1
**AT1G34410***	ARF21	0	-	-	-	-	0	1
**AT1G34390***	ARF22	0	-	-	-	-	0	1
**AT1G43950***	ARF23	0	-	-	-	-	0	1
**AT4G14560**	IAA1	1	-	-	-	-	-0.03	9.48E-01
**AT3G23030**	IAA2	2	-	+	-	-	-0.76	2.39E-21
**AT1G04240**	SHY2	2	-	-	-	-	3.22	4.66E-121
**AT5G43700**	ATAUX2-11	1	-	+	-	-	-0.45	2.15E-05
**AT1G15580***	IAA5	0	-	-	-	-	0	1
**AT1G52830**	IAA6	2	-	+	-	-	0	1
**AT3G23050**	IAA7	1	-	-	-	+	0.57	4.02E-07
**AT2G22670**	IAA8	2	-	+	-	-	1.39	6.40E-43
**AT5G65670**	IAA9	2	-	-	-	-	0.12	1.41E-01
**AT1G04100***	IAA10	0	-	-	-	-	-1.71	2.57E-14
**AT4G28640**	IAA11	0	-	-	-	-	-0.68	1.26E-02
**AT1G04550**	IAA12	1	-	-	-	-	-0.56	1.27E-02
**AT2G33310**	IAA13	1	-	+	-	-	-1.00	2.41E-15
**AT4G14550**	IAA14	2	-	-	-	-	-0.58	6.37E-03
**AT1G80390**	IAA15	0	-	-	-	+	0	1
**AT3G04730**	IAA16	1	-	+	-	-	-0.39	2.49E-09
**AT1G04250**	AXR3	1	-	-	-	+	0.67	1.70E-04
**AT1G51950**	IAA18	3	-	+	-	-	-0.75	2.02E-11
**AT3G15540**	IAA19	2	+	-	-	+	1.44	1.10E-03
**AT2G46990**	IAA20	1	-	-	-	+	1.93	4.30E-16
**AT3G16500**	PAP1	2	+	-	-	-	-1.48	1.32E-35
**AT4G29080**	PAP2	1	-	-	-	-	0.88	7.44E-07
**AT5G25890**	IAA28	0	-	+	-	-	-0.28	2.54E-01
**AT4G32280**	IAA29	0	-	-	-	-	1.97	3.36E-07
**AT3G62100**	IAA30	0	-	-	-	-	1.18	9.73E-04
**AT3G17600***	IAA31	0	-	-	-	-	0	1
**AT2G01200***	IAA32	0	-	-	-	-	2.88	8.13E-02
**AT1G15050***	IAA34	0	+	-	-	-	-0.44	4.35E-01
**AT4G03190**	AFB1	0	+	-	-	-	-1.55	3.32E-12
**AT3G26810**	AFB2	2	-	+	-	-	-0.15	3.61E-01
**AT1G12820**	AFB3	0	+	+	-	-	1.20	1.29E-40
**AT4G24390**	AFB4	0	-	-	-	-	0.58	1.03E-03
**AT5G49980**	AFB5	1	+	-	-	-	0.48	4.95E-05
**AT3G62980**	TIR1	1	-	+	-	-	-0.87	4.91E-18
**AT1G73590**	PIN1	1	+	+	-	-	0.13	7.71E-01
**AT5G57090**	PIN2	0	-	-	-	-	0.68	7.34E-02
**AT1G70940**	PIN3	2	+	+	-	-	-1.17	1.85E-27
**AT2G01420**	PIN4	3	+	+	+	-	0.36	2.92E-05
**AT5G16530***	PIN5	0	-	-	-	-	-0.98	7.92E-01
**AT1G77110**	PIN6	1	-	+	-	+	1.57	5.68E-02
**AT1G23080**	PIN7	2	-	-	-	-	-0.17	3.05E-01
**AT5G15100**	PIN8	0	-	-	-	+	0	1
**AT2G38120**	AUX1	2	-	-	-	+	0.91	3.36E-10
**AT5G01240**	LAX1	2	-	-	-	+	0.16	2.08E-01
**AT2G21050**	LAX2	0	-	-	-	-	0.46	1.45E-01
**AT1G77690**	LAX3	1	-	-	-	-	0.01	9.65E-01
**AT2G34650**	PID	2	-	-	-	+	0.35	1.04E-01
**AT2G26700**	PID2	0	-	-	-	-	-0.11	8.31E-01

Supplementary Table 2: Response of genes with activities in auxin signaling to WUS.

Adjusted p-value for RNA-seq data (WUS-GR, DEX vs. mock) was calculated using the Benjamini-Hochberg method in DESeq2. Asterisks denote genes in regions with closed chromatin[28]. ChIP-seq results for WUS binding, acetylation and methylation are given. Peaks column denotes number of WUS binding events at the locus; Ac denotes increase of acetylation at the locus, deAc denotes reduction in acetylation at the locus; Me denotes increase of methylation at the locus, deMe denotes reduction in methylation at the locus. Processed raw data including read counts can be found in Supplementary File 1.

AGI	Gene Name	WUS peaks	Ac	deAc	Me	deMe	Log2FC	p adj.
**AT3G28970**	AAR3	0	-	+	-	-	-0.15	6.05E-01
**AT2G36910**	ABCB1	1	-	-	-	-	0.29	1.89E-01
**AT1G10680**	ABCB10	1	-	+	-	+	0	1
**AT1G27940**	ABCB13	0	-	-	-	-	0	1
**AT1G28010**	ABCB14	0	+	+	-	-	0.22	3.46E-01
**AT3G28345**	ABCB15	0	-	-	-	+	2.29	2.67E-14
**AT3G28360**	ABCB16	0	-	-	+	+	0	1
**AT3G28380**	ABCB17	0	-	+	-	-	-0.69	8.20E-01
**AT3G28390**	ABCB18	0	-	-	-	+	0	1
**AT3G28860**	ABCB19	2	+	+	-	-	-0.55	4.02E-04
**AT4G25960**	ABCB2	0	+	-	-	-	0.75	3.93E-07
**AT3G55320**	ABCB20	0	-	-	-	-	0.48	8.69E-03
**AT3G62150**	ABCB21	0	-	-	-	-	-2.94	2.89E-108
**AT3G28415**	ABCB22	0	-	-	-	+	0	1
**AT5G03910**	ABCB29	0	-	+	-	-	-0.15	1.77E-01
**AT2G47000**	ABCB4	1	+	-	-	-	-0.26	3.71E-01
**AT2G39480**	ABCB6	2	-	-	-	-	-0.47	1.12E-03
**AT1G59870**	ABCG36	1	-	-	-	-	-1.90	3.99E-66
**AT3G53480**	ABCG37	0	+	-	-	-	-0.82	3.26E-03
**AT1G15520**	ABCG40	0	-	-	-	-	0.40	5.02E-02
**AT4G26080**	ABI1	0	-	+	-	-	-0.80	2.21E-18
**AT3G24650**	ABI3	1	+	-	-	+	1.14	4.23E-01
**AT2G05710**	ACO3	0	-	+	-	-	-0.82	6.22E-18
**AT2G22810**	ACS4	1	-	-	-	+	0	1
**AT4G11280**	ACS6	3	-	-	-	-	-0.49	5.50E-04
**AT5G09810**	ACT7	2	+	-	-	-	0.09	2.64E-01
**AT4G16420**	ADA2B	0	+	-	-	-	0.22	1.55E-02
**AT1G69260**	AFP1	1	-	-	-	-	0.55	6.83E-02
**AT5G13300**	AGD3	1	-	+	-	-	0.32	1.14E-03
**AT5G13790**	AGL15	1	-	-	-	-	-1.01	8.44E-05
**AT1G48410**	AGO1	1	-	-	-	+	0.49	2.22E-07
**AT2G35270**	AHL21	0	-	-	-	-	-1.04	3.21E-01
**AT2G46510**	AIB	1	-	-	-	-	0.38	1.73E-02
**AT5G57390**	AIL5	0	-	-	-	+	-0.04	9.04E-01
**AT5G10510**	AIL6	1	-	-	-	+	-0.55	2.46E-01
**AT5G65510**	AIL7	1	-	-	-	-	-0.21	9.10E-01
**AT4G12550**	AIR1	0	-	-	-	+	-5.02	6.48E-03
**AT3G07390**	AIR12	2	-	-	-	-	1.13	9.55E-24
**AT2G04160**	AIR3	2	-	-	-	+	-1.50	1.00E-13
**AT2G34680**	AIR9	0	-	-	-	-	-0.05	8.38E-01
**AT1G54100**	ALDH7B4	1	-	+	-	-	1.12	2.98E-82
**AT2G17800**	ARAC1	0	-	-	-	-	-0.05	8.48E-01
**AT1G59750**	ARF1	0	-	+	-	-	-0.09	4.26E-01
**AT2G28350**	ARF10	2	+	-	-	-	0.85	9.49E-04
**AT4G30080**	ARF16	0	+	-	-	-	0.14	6.29E-01
**AT1G77850**	ARF17	0	+	-	-	-	0.87	4.19E-05
**AT1G19220**	ARF19	0	+	-	-	-	1.12	2.96E-09
**AT2G33860**	ARF3	1	+	-	-	-	0.78	2.11E-06
**AT1G19850**	ARF5	1	-	-	-	-	0.86	1.25E-04
**AT1G30330**	ARF6	5	+	+	-	-	0.29	1.40E-02
**AT5G37020**	ARF8	1	-	-	-	-	-1.58	8.52E-20
**AT3G59900**	ARGOS	1	-	-	-	-	-4.69	4.35E-18
**AT2G37630**	AS1	1	+	-	-	-	0.30	4.03E-01
**AT5G05730**	ASA1	1	-	-	-	-	-1.38	6.94E-19
**AT1G54060**	ASIL1	0	-	-	-	-	-0.16	3.42E-01
**AT4G18710**	ASK7	3	-	-	-	-	-0.92	2.63E-35
**AT5G19140**	ATAILP1	2	-	-	-	-	0.69	2.82E-15
**AT3G01220**	ATHB-20	0	-	+	-	+	-0.29	7.71E-01
**AT5G66700**	ATHB-53	1	-	-	-	-	-2.31	1.35E-06
**AT4G32880**	ATHB-8	0	-	+	-	+	-0.50	1.44E-02
**AT5G27420**	ATL31	1	-	-	-	-	-0.32	5.80E-03
**AT2G46070**	ATMPK12	0	-	-	-	-	0.77	8.82E-08
**AT4G21440**	ATMYB102	2	-	-	-	+	0.24	5.42E-01
**AT5G15310**	ATMYB16	1	-	-	-	-	1.70	6.56E-29
**AT2G47190**	ATMYB2	1	-	-	-	-	-0.70	5.42E-03
**AT1G57560**	AtMYB50	0	-	-	-	-	0	1
**AT4G05100**	AtMYB74	2	-	-	+	-	-0.35	6.17E-01
**AT1G34670**	AtMYB93	0	-	-	+	-	2.03	3.11E-01
**AT2G26740**	ATSEH	1	-	-	-	-	-1.97	5.66E-95
**AT3G22650**	ATSFL61	0	-	-	-	-	0	1
**AT2G38120**	AUX1	2	-	-	-	+	0.91	3.36E-10
**AT2G32410**	AXL1	1	-	-	-	-	-0.24	3.72E-01
**AT1G05180**	AXR1	0	-	+	-	-	-0.23	1.58E-02
**AT1G54990**	AXR4	1	-	+	-	-	-1.52	3.09E-06
**AT3G19580**	AZF2	1	-	-	-	-	1.53	4.36E-16
**AT1G27740**	BHLH54	0	-	+	-	-	0	1
**AT4G33880**	BHLH85	0	-	-	-	-	0	1
**AT3G02260**	BIG	1	-	-	-	+	-0.26	5.70E-01
**AT2G35940**	BLH1	4	-	+	-	-	-1.72	2.08E-30
**AT2G01950**	BRL2	0	-	-	-	-	-0.03	9.11E-01

AGI	Gene Name	WUS peaks	Ac	deAc	Me	deMe	Log2FC	p adj.
**AT5G63160**	BT1	4	-	-	-	-	-0.33	1.01E-03
**AT3G48360**	BT2	3	+	-	-	-	0.45	4.86E-09
**AT5G67480**	BT4	1	-	-	-	-	1.37	3.24E-30
**AT4G37610**	BT5	2	+	-	-	-	1.26	1.14E-21
**AT2G02560**	CAND1	0	-	+	-	-	0.25	4.26E-02
**AT5G01490**	CAX4	0	-	-	+	+	-2.48	2.56E-03
**AT4G17615**	CBL1	2	-	-	-	-	-1.32	1.65E-14
**AT2G46830**	CCA1	0	-	-	-	-	-0.78	1.23E-10
**AT4G32810**	CCD8	0	-	-	-	-	-0.72	7.05E-02
**AT2G20000**	CDC27B	0	-	+	-	-	0.61	2.63E-06
**AT3G55120**	CHI1	1	-	-	-	-	0.76	1.46E-02
**AT3G07370**	CHIP	1	-	+	-	-	0.42	2.20E-06
**AT5G13930**	CHS	1	+	-	+	-	0.63	4.65E-01
**AT5G65940**	CHY1	0	-	-	-	-	-0.10	4.00E-01
**AT5G37770**	CML24	0	+	-	-	-	0.94	4.28E-04
**AT5G20990**	CNX1	0	-	-	-	-	-0.79	7.46E-22
**AT4G10100**	CNX7	0	+	-	-	-	-0.99	2.03E-08
**AT1G18890**	CPK10	0	+	+	-	-	0.22	3.83E-02
**AT3G57530**	CPK32	3	-	-	-	-	-0.51	6.10E-08
**AT2G17290**	CPK6	2	+	-	-	-	0.32	2.70E-03
**AT5G01270**	CPL2	1	-	-	-	-	0.16	3.04E-01
**AT1G22920**	CSN5A	1	-	+	-	-	-0.54	1.59E-06
**AT1G71230**	CSN5B	0	-	-	-	-	2.56	2.36E-03
**AT4G02570**	CUL1	0	+	-	-	-	-0.26	1.44E-03
**AT2G26710**	CYP734A1	2	+	-	-	-	1.32	6.53E-29
**AT5G07990**	CYP75B1	1	+	+	-	-	0.47	3.03E-01
**AT2G27690**	CYP94C1	0	-	-	-	-	-0.30	1.61E-01
**AT4G03400**	DFL2	2	-	+	-	-	-3.71	1.80E-83
**AT3G50410**	DOF3.4	0	-	+	-	-	-1.09	1.64E-03
**AT1G09700**	DRB1	0	-	-	-	-	0.82	1.29E-03
**AT5G22220**	E2FB	0	-	-	-	-	-0.04	8.48E-01
**AT5G14960**	E2FD	0	-	-	-	-	1.65	3.47E-08
**AT4G14430**	ECI2	1	-	+	-	-	0.36	4.75E-03
**AT2G19560**	EER5	0	-	-	-	-	-0.03	8.60E-01
**AT1G73730**	EIL3	0	-	-	-	-	0.55	6.23E-05
**AT3G20770**	EIN3	0	-	-	-	-	0.23	4.34E-03
**AT2G25930**	ELF3	0	-	-	-	-	0.31	3.39E-03
**AT5G13680**	ELP1	0	+	-	-	-	0.20	2.61E-01
**AT3G11220**	ELP4	0	+	-	-	-	0.12	6.05E-01
**AT1G28370**	ERF11	1	+	-	-	-	2.24	2.86E-27
**AT2G44840**	ERF13	1	-	+	-	+	2.11	9.22E-13
**AT1G24590**	ESR2	0	+	-	-	+	-1.42	4.50E-01
**AT1G66340**	ETR1	0	-	-	-	-	-0.30	9.46E-04
**AT1G27340**	FBX6	1	-	+	-	-	-2.18	7.88E-41
**AT1G26870**	FEZ	0	+	-	-	-	0	1
**AT5G08640**	FLS1	0	-	-	-	-	1.95	1.51E-01
**AT5G54500**	FQR1	2	-	-	-	-	0.89	1.04E-16
**AT3G26790**	FUS3	0	-	+	-	-	2.16	9.74E-02
**AT2G46270**	GBF3	0	+	-	-	-	0.63	1.33E-07
**AT1G61120**	GES	0	-	-	-	-	-2.64	1.24E-03
**AT2G39550**	GGB	1	-	-	-	-	-0.32	1.45E-02
**AT2G14960**	GH3.1	0	-	-	-	+	-0.28	9.15E-01
**AT1G28130**	GH3.17	2	-	-	-	-	-0.39	9.13E-03
**AT2G23170**	GH3.3	0	-	+	-	-	-0.93	1.67E-05
**AT1G59500**	GH3.4	0	-	-	-	-	0	1
**AT4G27260**	GH3.5	3	-	-	-	-	-0.48	4.82E-07
**AT5G54510**	GH3.6	1	-	+	-	-	2.81	2.19E-46
**AT2G47750**	GH3.9	0	-	-	-	-	0.96	9.34E-02
**AT3G63010**	GID1B	3	-	-	-	-	-1.83	2.95E-51
**AT4G32690**	GLB3	1	-	-	-	-	-1.08	7.55E-08
**AT4G03190**	GRH1	0	+	-	-	-	-1.55	3.32E-12
**AT1G28480**	GRXC9	1	-	-	-	-	1.82	1.35E-17
**AT5G50320**	HAG3	0	+	+	-	-	-0.46	2.86E-05
**AT5G47370**	HAT2	3	-	-	-	-	0.31	1.55E-01
**AT4G16780**	HAT4	3	-	-	-	-	-1.77	3.79E-24
**AT4G36740**	HB-5	0	-	+	-	-	2.90	6.73E-07
**AT4G37580**	HLS1	2	+	-	-	-	-0.26	3.16E-01
**AT5G45710**	HSFA4C	0	-	-	-	-	0.08	6.97E-01
**AT4G14560**	IAA1	1	-	-	-	-	-0.03	9.48E-01
**AT1G04100**	IAA10	0	-	-	-	-	-1.71	2.57E-14
**AT4G28640**	IAA11	0	-	-	-	-	-0.68	1.26E-02
**AT1G04550**	IAA12	1	-	-	-	-	-0.56	1.27E-02
**AT2G33310**	IAA13	1	-	+	-	-	-1.00	2.41E-15
**AT4G14550**	IAA14	2	-	-	-	-	-0.58	6.37E-03
**AT1G80390**	IAA15	0	-	-	-	+	0	1
**AT3G04730**	IAA16	1	-	+	-	-	-0.39	2.49E-09
**AT1G04250**	IAA17	1	-	-	-	+	0.67	1.70E-04
**AT1G51950**	IAA18	3	-	+	-	-	-0.75	2.02E-11
**AT3G15540**	IAA19	2	+	-	-	+	1.44	1.10E-03
**AT3G23030**	IAA2	2	-	+	-	-	-0.76	2.39E-21

AGI	Gene Name	WUS peaks	Ac	deAc	Me	deMe	Log2FC	p adj.
**AT2G46990**	IAA20	1	-	-	-	+	1.93	4.30E-16
**AT3G16500**	IAA26	2	+	-	-	-	-1.48	1.32E-35
**AT4G29080**	IAA27	1	-	-	-	-	0.88	7.44E-07
**AT5G25890**	IAA28	0	-	+	-	-	-0.28	2.54E-01
**AT4G32280**	IAA29	0	-	-	-	-	1.97	3.36E-07
**AT3G62100**	IAA30	0	-	-	-	-	1.18	9.73E-04
**AT3G17600**	IAA31	0	-	-	-	-	0	1
**AT2G01200**	IAA32	0	-	-	-	-	2.88	8.13E-02
**AT5G57420**	IAA33	0	-	+	-	+	0	1
**AT1G15050**	IAA34	0	+	-	-	-	-0.44	4.35E-01
**AT5G43700**	IAA4	1	-	+	-	-	-0.45	2.15E-05
**AT1G15580**	IAA5	0	-	-	-	-	0	1
**AT1G52830**	IAA6	2	-	+	-	-	0	1
**AT3G23050**	IAA7	1	-	-	-	+	0.57	4.02E-07
**AT2G22670**	IAA8	2	-	+	-	-	1.39	6.40E-43
**AT5G65670**	IAA9	2	-	-	-	-	0.12	1.41E-01
**AT2G04550**	IBR5	0	-	-	-	-	0.28	1.84E-02
**AT1G47510**	IP5P11	1	-	-	-	-	-1.90	6.10E-08
**AT4G18010**	IP5P2	4	-	-	-	-	-1.04	3.42E-12
**AT2G22240**	IPS2	0	+	-	-	-	1.13	2.29E-14
**AT4G19690**	IRT1	0	-	-	-	-	0	1
**AT2G46370**	JAR1	2	-	-	-	-	-0.76	6.78E-13
**AT5G13220**	JAZ10	1	-	-	-	-	2.12	4.86E-30
**AT1G19640**	JMT	2	-	-	-	-	0	1
**AT5G49720**	KOR	1	-	+	-	-	-0.35	2.80E-07
**AT1G77690**	LAX3	1	-	-	-	-	0.01	9.65E-01
**AT3G58190**	LBD29	0	-	-	-	-	0	1
**AT1G01060**	LHY	1	-	-	-	-	-0.20	6.39E-01
**AT5G06300**	LOG7	5	-	+	-	-	0.01	9.73E-01
**AT5G12330**	LRP1	2	+	-	-	+	0.09	8.26E-01
**AT4G20380**	LSD1	0	+	-	-	-	0.39	2.24E-04
**AT2G39370**	MAKR4	0	-	-	-	-	0	1
**AT3G09940**	MDAR3	0	-	-	-	+	-2.00	4.29E-02
**AT1G74660**	MIF1	0	-	-	-	-	1.14	3.39E-03
**AT1G10210**	MPK1	0	+	-	-	-	-0.16	3.04E-01
**AT1G01560**	MPK11	0	+	-	-	-	2.01	1.50E-40
**AT4G01370**	MPK4	0	-	+	-	-	0.28	7.86E-03
**AT2G43790**	MPK6	1	-	-	-	-	-0.91	3.41E-29
**AT3G06490**	MYB108	2	-	-	-	-	-2.35	2.86E-09
**AT3G55730**	MYB109	1	-	-	-	-	1.12	4.18E-05
**AT2G47460**	MYB12	1	-	-	-	+	0	1
**AT2G31180**	MYB14	0	-	-	-	+	0	1
**AT3G23250**	MYB15	2	-	-	-	+	-2.72	1.40E-16
**AT1G22640**	MYB3	2	-	+	-	-	-1.50	3.49E-16
**AT3G28910**	MYB30	3	-	-	-	-	1.26	4.36E-16
**AT5G67300**	MYB44	3	-	-	-	-	1.45	6.93E-22
**AT1G18570**	MYB51	3	-	+	-	+	-0.88	1.61E-08
**AT5G59780**	MYB59	2	-	-	-	-	-1.03	2.21E-02
**AT1G09540**	MYB61	1	-	-	-	-	-0.89	5.37E-01
**AT1G56650**	MYB75	1	-	-	-	-	1.83	1.39E-01
**AT5G07700**	MYB76	1	-	+	-	-	0	1
**AT3G47600**	MYB94	1	-	-	-	+	0.64	2.08E-04
**AT1G52890**	NAC019	2	-	+	-	-	-0.21	3.78E-01
**AT1G56010**	NAC021	2	-	-	-	-	-0.47	1.24E-01
**AT3G15500**	NAC055	2	+	-	-	+	-0.13	3.37E-01
**AT5G39610**	NAC92	2	-	+	-	-	-0.15	3.46E-01
**AT5G63310**	NDPK2	0	-	-	-	-	0.68	1.17E-10
**AT2G34720**	NFYA4	1	+	-	-	+	0.11	5.46E-01
**AT1G31880**	NLM9	1	-	-	-	-	1.44	2.45E-10
**AT1G32450**	NPF7.3	0	-	+	-	-	-0.41	2.46E-01
**AT5G20730**	NPH4	0	+	-	-	-	0.17	2.46E-01
**AT1G80680**	NUP96	0	-	+	-	-	0.06	7.11E-01
**AT2G06050**	OPR3	1	+	-	-	-	0.91	1.13E-20
**AT5G54490**	PBP1	0	-	-	-	-	0.84	4.32E-03
**AT5G64890**	PEP2	0	-	-	-	-	-4.68	2.22E-02
**AT5G56290**	PEX5	1	-	-	-	-	-0.28	6.52E-03
**AT5G40770**	PHB3	0	-	-	-	-	-0.33	5.54E-04
**AT2G34650**	PID	2	-	-	-	+	0.35	1.04E-01
**AT2G43010**	PIF4	2	-	-	-	-	1.79	1.26E-75
**AT3G59060**	PIF5	3	-	-	-	-	-0.75	1.01E-22
**AT1G73590**	PIN1	1	+	+	-	-	0.13	7.71E-01
**AT5G57090**	PIN2	0	-	-	-	-	0.68	7.34E-02
**AT1G70940**	PIN3	2	+	+	-	-	-1.17	1.85E-27
**AT5G15100**	PIN8	0	-	-	-	+	0	1
**AT2G25170**	PKL	0	-	+	-	-	0.27	1.60E-02
**AT2G19690**	PLA2-BETA	0	+	-	-	-	2.18	2.28E-01
**AT3G05630**	PLPZETA2	1	+	-	-	-	-1.43	1.83E-05
**AT4G39403**	PLS	3	-	-	-	-	3.03	2.12E-01
**AT1G25490**	PP2AA1	0	+	+	-	-	-0.27	5.88E-03
**AT4G29940**	PRH	1	+	+	-	-	-0.13	2.15E-01

AGI	Gene Name	WUS peaks	Ac	deAc	Me	deMe	Log2FC	p adj.
**AT3G62680**	PRP3	0	-	+	-	+	0	1
**AT3G21110**	PUR7	0	-	-	-	-	0.21	3.13E-01
**AT2G41820**	PXC3	2	-	-	-	-	-1.78	1.49E-03
**AT1G06400**	RABA1A	0	+	-	-	-	-0.09	5.72E-01
**AT1G64060**	RBOHF	2	+	+	-	-	0.09	7.26E-01
**AT1G32230**	RCD1	1	-	+	-	-	-0.61	2.11E-09
**AT4G36800**	RCE1	1	-	-	-	-	-0.61	6.63E-12
**AT4G27410**	RD26	0	+	-	-	-	2.38	1.17E-113
**AT2G01570**	RGA	3	+	+	-	-	-2.17	1.11E-44
**AT3G26090**	RGS1	0	-	-	-	-	-1.33	5.83E-19
**AT1G78570**	RHM1	1	+	+	-	-	0.41	4.09E-03
**AT5G20570**	ROC1	0	-	+	-	-	-0.39	4.95E-04
**AT1G69270**	RPK1	1	-	-	-	-	-1.64	3.08E-05
**AT3G53020**	RPL24B	0	+	-	-	-	-0.50	4.28E-05
**AT4G38630**	RPN10	1	-	+	-	-	-0.23	4.87E-03
**AT1G64520**	RPN12A	1	-	-	-	-	-0.30	7.15E-03
**AT3G14050**	RSH2	1	-	+	-	-	0.23	3.32E-02
**AT1G31340**	RUB1	1	-	-	-	-	-1.02	1.11E-16
**AT5G17300**	RVE1	1	-	+	-	-	-1.13	1.26E-12
**AT5G37260**	RVE2	1	-	-	-	-	1.64	1.26E-29
**AT5G02840**	RVE4	0	-	-	-	-	-0.84	1.25E-10
**AT4G01280**	RVE5	0	-	-	-	-	0.22	9.55E-02
**AT3G09600**	RVE8	1	-	-	-	-	-1.21	7.36E-20
**AT5G59220**	SAG113	4	-	-	-	+	-1.48	5.36E-03
**AT3G28210**	SAP12	1	-	+	-	-	0.72	2.18E-10
**AT1G33410**	SAR1	0	+	-	-	-	0.24	6.46E-02
**AT4G38850**	SAUR15	1	-	-	-	-	0	1
**AT5G18010**	SAUR19	1	-	-	-	-	0	1
**AT5G18020**	SAUR20	1	-	-	-	-	0	1
**AT5G18030**	SAUR21	1	-	-	-	-	0.08	9.64E-01
**AT5G18050**	SAUR22	0	-	-	-	-	0	1
**AT5G18060**	SAUR23	0	-	-	-	-	0	1
**AT5G18080**	SAUR24	1	-	-	-	-	0.57	8.41E-01
**AT3G03830**	SAUR28	1	-	+	-	-	0	1
**AT2G46690**	SAUR32	1	-	-	-	-	-0.86	1.06E-06
**AT2G45210**	SAUR36	2	-	-	-	+	-1.82	9.59E-14
**AT1G79130**	SAUR40	0	-	-	-	-	-2.95	2.72E-01
**AT1G16510**	SAUR41	1	-	-	-	-	0.41	7.28E-01
**AT4G34760**	SAUR50	1	-	-	-	-	-1.83	1.42E-02
**AT1G29430**	SAUR62	0	-	-	-	-	0	1
**AT1G29440**	SAUR63	1	-	-	-	-	-4.15	7.16E-02
**AT1G29450**	SAUR64	0	-	-	-	-	-1.41	3.27E-01
**AT1G29500**	SAUR66	1	-	-	-	-	-0.98	5.70E-01
**AT1G29510**	SAUR67	0	-	-	-	-	-5.26	6.53E-04
**AT1G29490**	SAUR68	0	-	-	-	-	0	1
**AT1G56150**	SAUR71	1	-	-	-	-	1.63	4.98E-02
**AT3G12830**	SAUR72	2	-	-	-	-	0.47	3.51E-01
**AT4G05530**	SDRA	0	-	-	-	-	-0.54	1.33E-04
**AT4G23570**	SGT1A	1	-	-	-	-	-0.38	1.03E-08
**AT4G11260**	SGT1B	0	-	+	-	-	-0.62	2.20E-14
**AT1G04240**	SHY2	2	-	-	-	-	3.22	4.66E-121
**AT1G16540**	SIR3	0	-	-	-	-	0.02	9.13E-01
**AT1G21410**	SKP2A	0	+	-	-	-	0.74	1.99E-07
**AT4G13520**	SMAP1	1	-	-	-	-	-0.31	2.47E-02
**AT3G24280**	SMAP2	0	-	-	-	-	0	1
**AT4G16890**	SNC1	1	+	+	-	-	0.06	6.58E-01
**AT1G74100**	SOT16	0	+	-	-	-	0.41	1.82E-01
**AT4G34710**	SPE2	4	-	+	-	-	-0.58	1.69E-03
**AT1G28560**	SRD2	0	-	-	-	-	0.37	5.95E-01
**AT3G11820**	SYP121	1	-	+	-	-	0.43	7.88E-07
**AT3G52400**	SYP122	1	-	-	-	-	-0.31	5.37E-02
**AT2G24850**	TAT3	0	-	-	-	-	0.93	5.57E-01
**AT1G53230**	TCP3	1	-	-	-	-	-0.19	8.18E-01
**AT3G16640**	TCTP1	0	-	-	-	-	-0.29	1.07E-02
**AT5G06960**	TGA5	0	-	-	-	-	0.60	8.92E-06
**AT2G34600**	TIFY	1	-	-	-	-	-0.28	2.89E-01
**AT1G19180**	TIFY10A	2	-	-	-	-	1.45	1.83E-18
**AT1G74950**	TIFY10B	1	-	-	-	-	-0.83	1.73E-22
**AT1G17380**	TIFY11A	1	+	-	-	-	1.46	3.54E-11
**AT3G62980**	TIR1	1	-	+	-	-	-0.87	4.91E-18
**AT1G15750**	TPL	1	+	-	-	-	-0.30	2.25E-03
**AT1G08030**	TPST	1	+	-	-	-	-0.05	7.43E-01
**AT1G49950**	TRB1	0	-	-	-	-	0.23	3.47E-02
**AT5G67580**	TRB2	0	+	-	-	-	1.04	1.93E-28
**AT3G49850**	TRB3	0	-	-	-	-	-0.02	9.45E-01
**AT5G59430**	TRP1	1	-	+	-	-	0.96	4.91E-20
**AT5G42980**	TRX3	0	-	-	-	-	-0.47	7.51E-10
**AT5G24520**	TTG1	1	+	-	-	-	-0.45	6.43E-06
**AT2G42580**	TTL3	1	+	-	-	-	-0.12	2.04E-01
**AT3G20830**	UCNL	2	+	-	-	+	1.28	1.88E-08

AGI	Gene Name	WUS peaks	Ac	deAc	Me	deMe	Log2FC	p adj.
AT4G00080	UNE11	0	-	-	-	-	0	1
AT3G63300	VAB	0	-	+	-	-	-2.48	3.84E-15
AT3G13300	VCS	0	-	-	-	-	0.11	4.17E-01
AT1G14000	VIK	0	-	+	-	-	0.22	7.50E-02
AT5G55120	VTC5	1	+	-	-	-	1.37	2.91E-23
AT3G11260	WOX5	0	-	-	-	+	-1.19	5.39E-01
AT2G47260	WRKY23	2	+	+	-	-	-0.68	6.96E-02
AT5G57740	XBAT32	0	-	+	-	-	0.92	1.18E-03
AT4G34390	XLG2	0	+	-	-	-	0.10	5.00E-01
AT5G57560	XTH22	3	+	-	-	-	-0.30	5.39E-02
AT2G06850	XTH4	2	-	-	-	-	-0.47	5.33E-08
AT4G37390	YDK1	1	-	-	-	-	3.61	3.69E-169
AT5G51640	YLS7	0	-	-	-	-	0.22	3.22E-01
AT1G27730	ZAT10	3	+	-	-	-	-1.81	1.46E-35
AT5G51470		0	-	+	+	+	0	1
AT3G20220		0	-	-	-	-	0	1
AT2G37030		0	-	-	-	-	1.35	4.92E-01
AT4G34810		0	-	-	-	-	-6.84	6.66E-14
AT1G19840		0	+	-	-	-	1.30	5.24E-01
AT4G34770		1	-	-	-	-	-5.89	8.70E-10
AT1G63720		1	+	-	-	-	0.68	9.03E-05
AT3G43120		0	-	-	-	-	0	1
AT1G19830		0	-	-	-	+	-3.36	6.43E-02
AT4G38840		1	-	-	-	-	-3.31	2.26E-03
AT3G26760		0	-	+	-	+	-1.92	2.90E-17
AT1G48670		0	-	+	-	-	0	1
AT1G17520		0	-	-	-	-	-0.18	1.36E-01
AT1G70000		1	-	-	-	-	-2.22	1.93E-11
AT5G20820		0	-	-	-	-	0	1
AT3G51200		0	-	-	-	-	0	1
AT5G13380		0	-	-	-	-	0	1
AT2G21210		2	-	-	-	-	-0.47	4.87E-01
AT5G42410		0	-	-	-	-	0	1
AT1G17345		2	-	-	-	-	-1.15	2.31E-02
AT1G74840		0	-	-	-	-	1.11	8.83E-35
AT3G03840		0	-	-	-	-	0	1
AT1G76190		0	-	-	-	-	0	1
AT1G15430		1	-	+	-	-	-0.19	2.29E-01
AT4G33940		1	-	-	-	-	0.53	2.59E-07
AT2G21200		1	-	-	-	-	0.73	7.99E-01
AT3G25880		1	-	-	-	-	-1.92	8.85E-02
AT1G49010		1	-	+	-	-	-1.59	4.17E-06
AT1G48660		0	-	-	-	-	0	1
AT4G18950		1	-	+	-	-	0.61	3.38E-06
AT4G34780		0	-	+	-	-	0	1
AT5G13350		0	-	-	-	-	0	1
AT2G36210		0	+	-	-	-	0.93	3.14E-01
AT2G18010		1	-	-	-	+	0	1
AT1G75590		0	-	-	-	-	0.40	8.58E-01
AT5G50120		0	-	-	-	-	0	1
AT2G24400		0	-	-	-	-	-0.60	2.35E-01
AT3G61900		0	-	-	-	-	-0.70	1.41E-01
AT1G43040		0	-	-	-	-	0	1
AT2G21220		1	-	-	-	-	-0.32	9.19E-01
AT4G34790		1	-	-	-	-	-5.25	3.70E-04
AT3G03850		0	-	-	-	-	0	1
AT4G12410		0	-	-	-	-	-0.77	4.08E-01
AT4G00880		1	-	-	-	+	-0.91	2.56E-01
AT4G13790		0	-	-	-	-	0	1
AT4G09530		0	-	-	-	+	0	1
AT5G38895		0	-	-	-	-	0.09	5.32E-01
AT3G60690		3	-	-	-	-	-2.32	2.18E-09
AT4G31320		0	-	-	-	-	0	1
AT3G09980		1	-	-	-	-	0.85	9.78E-16
AT5G10990		0	+	-	-	-	-0.18	9.60E-01
AT5G53590		1	-	-	-	-	1.10	1.17E-02
AT4G36110		0	-	-	-	+	-0.04	9.56E-01
AT5G66260		0	-	-	-	-	0	1
AT4G22620		2	-	-	-	-	2.38	1.16E-01
AT5G50760		0	+	-	-	-	0.99	1.60E-02
AT3G09870		0	-	-	-	-	0	1
AT3G16350		0	+	-	-	-	-0.62	8.84E-02
AT1G63840		2	-	+	-	-	-0.84	1.91E-14
AT4G35030		0	-	-	+	-	1.46	2.20E-02
AT3G03847		1	-	-	-	-	0	1
AT1G20470		0	-	-	-	-	0.28	8.72E-01
AT3G03820		0	-	-	-	-	0	1
AT4G38860		2	-	-	-	-	1.83	1.27E-01
AT1G78100		4	+	-	-	-	1.72	2.64E-13
AT4G15430		1	-	+	-	-	-0.86	7.50E-02

AGI	Gene Name	WUS peaks	Ac	deAc	Me	deMe	Log2FC	p adj.
AT5G20810		0	-	-	-	+	2.68	1.42E-01
AT2G28085		0	-	-	+	-	0	1
AT2G28780		0	-	-	-	-	-1.96	1.01E-01
AT3G53250		0	+	-	-	-	-0.87	4.57E-01
AT1G29460		0	-	-	-	-	-1.63	2.59E-01
AT2G25790		1	-	-	-	+	-0.37	4.07E-03
AT3G12955		0	-	-	-	+	3.97	1.40E-02
AT4G25030		1	-	-	-	-	-2.87	4.89E-254
AT1G78990		0	-	-	-	-	3.66	1.99E-01
AT4G37295		1	-	-	-	-	2.44	6.80E-09
AT1G29420		1	-	-	-	-	0	1
AT5G27780		0	-	-	-	-	0	1
AT5G13370		1	-	-	-	-	0.70	9.58E-13
AT5G03310		1	-	-	-	-	0	1
AT1G72430		2	+	-	-	-	0.63	1.40E-03
AT4G26400		0	+	-	-	-	0.04	7.06E-01
AT1G23160		0	-	-	-	+	-4.47	3.10E-05
AT5G13360		1	-	-	-	-	-1.23	3.64E-08
AT5G63190		0	+	-	-	-	0.48	3.00E-08
AT2G16580		0	-	-	-	-	-1.66	4.98E-01
AT4G34750		2	-	+	-	-	-1.25	2.55E-03
AT1G75580		2	-	-	-	-	-1.85	6.28E-04
AT1G48690		0	-	-	-	+	0	1
AT4G34800		1	-	+	-	-	-6.31	4.05E-05

Supplementary Table 3: Response of genes with annotated activities in "auxin response" to WUS.

Adjusted p-value for RNA-seq data (WUS-GR, DEX vs. mock) was calculated using the Benjamini-Hochberg method in DESeq2. ChIP-seq results for WUS binding, acetylation and methylation are given. Peaks column denotes number of WUS binding events at the locus; Ac denotes increase of acetylation at the locus, deAc denotes reduction in acetylation at the locus; Me denotes increase of methylation at the locus, deMe denotes reduction in methylation at the locus. Processed raw data including read counts can be found in Supplementary File 1.

Supplementary Data 1: Processed raw data of genomic datasets.

Supplementary Data 2: Interactive HTML file allowing intersections of genomic datasets.
