# Supplementary material for: WUSCHEL acts as an auxin response rheostat to maintain apical stem cells in Arabidopsis
Source: Nat Commun. 2019 Nov 8;10:5093. doi: 10.1038/s41467-019-13074-9 (PMC6841675; doi:10.1038/s41467-019-13074-9)
Supplement: Supplementary file 3 — Description of Additional Supplementary Files [file 41467_2019_13074_MOESM3_ESM.pdf]

Description of Additional Supplementary Files

File Name: Supplementary Data 1
Description: Processed raw data of genomic datasets

File Name: Supplementary Data 2
Description: Interactive HTML file allowing intersections of genomic datasets.
