# Supplementary material for: WUSCHEL acts as an auxin response rheostat to maintain apical stem cells in Arabidopsis
Source: Nat Commun. 2019 Nov 8;10:5093. doi: 10.1038/s41467-019-13074-9 (PMC6841675; doi:10.1038/s41467-019-13074-9)
Supplement: Supplementary file 5 — Supplementary Data 2 [file 41467_2019_13074_MOESM5_ESM.html]

Interactive tool for exploring genomic data from WUSCHEL acts as a rheostat on the auxin pathway to maintain apical stem cells in Arabidopsis


# Interactive tool for exploring genomic data from “WUSCHEL acts as a rheostat on the auxin pathway to maintain apical stem cells in Arabidopsis”

#### *Olga Ermakova*

- Description
- Venn WUS binding and Acetylation
  - Table with genes
  - Venn Plot
- Venn WUS binding and Methylation
  - Table with genes
  - Venn Plot
- Venn WUS binding, transcriptional response
  - Table with genes
  - Venn Plot
- Venn WUS binding, transcriptional response, Deacetylation
  - Table with genes
  - Venn Plot
- Venn WUS binding, transcriptional response, genes repressed by WUS and derepressed by TSA
  - Table with genes
  - Venn Plot
- Venn WUS binding, genes repressed by WUS, genes derepressed by TSA and response to auxin
  - Table with genes
  - Venn Plot
- Session Info

# Description

Information is presented in the form of Venn diagrams and interactive tables. To get list of genes from specific subset of Venn diagram, you can use “set” filter in table. Overlaping subsets are separated by underscore (e.g. keyword “Acetylation\_Deacetylation” represents the genes having both Acetylation and Deacetylation marks). You can select multiple keywords to combine subsets. Using column “link” you will be redirected to the site of the corresponding gene at Arabidopsis.org. After filtering or selection you can use “Copy” or “Download” buttons to get the table.

# Venn WUS binding and Acetylation

Venn diagram for genes bound by WUSCHEL and having acetylation changes

## Table with genes

## Venn Plot

# Venn WUS binding and Methylation

Venn diagram for genes bound by WUSCHEL and having methylation changes

## Table with genes

## Venn Plot

# Venn WUS binding, transcriptional response

Venn plot for genes deregulated by WUSCHEL

## Table with genes

## Venn Plot

# Venn WUS binding, transcriptional response, Deacetylation

Venn plot for genes deregulated by WUSCHEL having deacetylation marks.

## Table with genes

## Venn Plot

# Venn WUS binding, transcriptional response, genes repressed by WUS and derepressed by TSA

Venn plot for genes having WUSCHEL binding site, deregulated by WUSCHEL, and set of genes derepressed by TSA.

## Table with genes

## Venn Plot

# Venn WUS binding, genes repressed by WUS, genes derepressed by TSA and response to auxin

Venn plot for genes having WUSCHEL binding site, repressed by WUSCHEL, genes derepressed by TSA and genes annotated to GO group “response to auxin”.

## Table with genes

## Venn Plot

# Session Info

```
## R version 3.5.0 (2018-04-23)
## Platform: x86_64-apple-darwin15.6.0 (64-bit)
## Running under: macOS  10.14.4
## 
## Matrix products: default
## BLAS: /Library/Frameworks/R.framework/Versions/3.5/Resources/lib/libRblas.0.dylib
## LAPACK: /Library/Frameworks/R.framework/Versions/3.5/Resources/lib/libRlapack.dylib
## 
## locale:
## [1] en_US.UTF-8/en_US.UTF-8/en_US.UTF-8/C/en_US.UTF-8/en_US.UTF-8
## 
## attached base packages:
## [1] stats4    parallel  stats     graphics  grDevices utils     datasets 
## [8] methods   base     
## 
## other attached packages:
##  [1] org.At.tair.db_3.6.0        AnnotationDbi_1.42.1       
##  [3] systemPipeR_1.14.0          ShortRead_1.38.0           
##  [5] GenomicAlignments_1.16.0    SummarizedExperiment_1.10.1
##  [7] DelayedArray_0.6.5          matrixStats_0.54.0         
##  [9] Biobase_2.40.0              BiocParallel_1.14.2        
## [11] Rsamtools_1.32.3            Biostrings_2.48.0          
## [13] XVector_0.20.0              GenomicRanges_1.32.6       
## [15] GenomeInfoDb_1.16.0         IRanges_2.14.11            
## [17] S4Vectors_0.18.3            BiocGenerics_0.26.0        
## [19] DT_0.4                      limma_3.36.3               
## 
## loaded via a namespace (and not attached):
##  [1] Category_2.46.0        bitops_1.0-6           bit64_0.9-7           
##  [4] RColorBrewer_1.1-2     progress_1.2.0         httr_1.3.1            
##  [7] rprojroot_1.3-2        Rgraphviz_2.24.0       tools_3.5.0           
## [10] backports_1.1.3        R6_2.4.0               DBI_1.0.0             
## [13] lazyeval_0.2.1         colorspace_1.3-2       tidyselect_0.2.5      
## [16] prettyunits_1.0.2      bit_1.1-14             compiler_3.5.0        
## [19] sendmailR_1.2-1        graph_1.58.0           rtracklayer_1.40.5    
## [22] scales_1.0.0           checkmate_1.8.5        BatchJobs_1.7         
## [25] genefilter_1.62.0      RBGL_1.56.0            stringr_1.4.0         
## [28] digest_0.6.18          rmarkdown_1.10         AnnotationForge_1.22.2
## [31] base64enc_0.1-3        pkgconfig_2.0.2        htmltools_0.3.6       
## [34] htmlwidgets_1.2        rlang_0.3.1            RSQLite_2.1.1         
## [37] shiny_1.1.0            BBmisc_1.11            GOstats_2.46.0        
## [40] jsonlite_1.5           hwriter_1.3.2          crosstalk_1.0.0       
## [43] dplyr_0.8.0.1          RCurl_1.95-4.11        magrittr_1.5          
## [46] GO.db_3.6.0            GenomeInfoDbData_1.1.0 Matrix_1.2-14         
## [49] Rcpp_1.0.0             munsell_0.5.0          stringi_1.3.1         
## [52] yaml_2.2.0             edgeR_3.22.3           zlibbioc_1.26.0       
## [55] plyr_1.8.4             grid_3.5.0             blob_1.1.1            
## [58] promises_1.0.1         crayon_1.3.4           lattice_0.20-35       
## [61] splines_3.5.0          GenomicFeatures_1.32.2 annotate_1.58.0       
## [64] hms_0.4.2              locfit_1.5-9.1         knitr_1.20            
## [67] pillar_1.3.1           rjson_0.2.20           biomaRt_2.36.1        
## [70] XML_3.98-1.16          glue_1.3.0             evaluate_0.11         
## [73] latticeExtra_0.6-28    data.table_1.11.4      httpuv_1.4.5          
## [76] gtable_0.2.0           purrr_0.3.0            assertthat_0.2.0      
## [79] ggplot2_3.1.0          mime_0.5               xtable_1.8-2          
## [82] later_0.7.3            survival_2.42-6        tibble_2.0.1          
## [85] pheatmap_1.0.10        memoise_1.1.0          brew_1.0-6            
## [88] GSEABase_1.42.0
```
