# Supplementary material for: WUSCHEL acts as an auxin response rheostat to maintain apical stem cells in Arabidopsis
Source: Nat Commun. 2019 Nov 8;10:5093. doi: 10.1038/s41467-019-13074-9 (PMC6841675; doi:10.1038/s41467-019-13074-9)
Supplement: Supplementary file 6 — Reporting Summary [file 41467_2019_13074_MOESM6_ESM.pdf]

nature research

Corresponding author(s): Jan U. Lohmann

Last updated by author(s): 2019/08/13

Reporting Summary

Nature Research wishes to improve the reproducibility of the work that we publish. This form provides structure for consistency and transparency in reporting. For further information on Nature Research policies, seeAuthors & Referees and theEditorial Policy Checklist .

Statistics

For all statistical analyses, confirm that the following items are present in the figure legend, table legend, main text, or Methods section.

n/a	Confirmed	
☐	☒	The exact sample size (*n*) for each experimental group/condition, given as a discrete number and unit of measurement
☐	☒	A statement on whether measurements were taken from distinct samples or whether the same sample was measured repeatedly
☐	☒	The statistical test(s) used AND whether they are one- or two-sided<br>*Only common tests should be described solely by name; describe more complex techniques in the Methods section.*
☒	☐	A description of all covariates tested
☐	☒	A description of any assumptions or corrections, such as tests of normality and adjustment for multiple comparisons
☒	☐	A full description of the statistical parameters including central tendency (e.g. means) or other basic estimates (e.g. regression coefficient) AND variation (e.g. standard deviation) or associated estimates of uncertainty (e.g. confidence intervals)
☒	☐	For null hypothesis testing, the test statistic (e.g. *F*, *t*, *r*) with confidence intervals, effect sizes, degrees of freedom and *P* value noted<br>*Give P values as exact values whenever suitable.*
☒	☐	For Bayesian analysis, information on the choice of priors and Markov chain Monte Carlo settings
☒	☐	For hierarchical and complex designs, identification of the appropriate level for tests and full reporting of outcomes
☒	☐	Estimates of effect sizes (e.g. Cohen's *d*, Pearson's *r*), indicating how they were calculated

Our web collection on statistics for biologists contains articles on many of the points above.

Software and code

Policy information about availability of computer code

Data collection	does not apply
Data analysis	does not apply

For manuscripts utilizing custom algorithms or software that are central to the research but not yet described in published literature, software must be made available to editors/reviewers. We strongly encourage code deposition in a community repository (e.g. GitHub). See the Nature Research guidelines for submitting code & software for further information.

Data

Policy information about availability of data

All manuscripts must include a data availability statement. This statement should provide the following information, where applicable:

- Accession codes, unique identifiers, or web links for publicly available datasets
- A list of figures that have associated raw data
- A description of any restrictions on data availability

Sequence data that support the findings of this study have been deposited in GEO under accession GSE122611. Image data are available upon request.

Field-specific reporting

Please select the one below that is the best fit for your research. If you are not sure, read the appropriate sections before making your selection.

☒ Life sciences ☐ Behavioural & social sciences ☐ Ecological, evolutionary & environmental sciences

For a reference copy of the document with all sections, see nature.com/documents/nr-reporting-summary-flat.pdf

Life sciences study design

All studies must disclose on these points even when the disclosure is negative.

Sample size	Sample size refers to number of individual plants analyzed.
Data exclusions	No data were excluded.
Replication	All experiments have been independently replicated at least twice.
Randomization	Control and experimental samples were grown, harvested and processed in parallel under identical conditions.
Blinding	For analysis of in situ hybridization experiments, samples were number-coded and blindly analyzed by independent scientists.

Reporting for specific materials, systems and methods

We require information from authors about some types of materials, experimental systems and methods used in many studies. Here, indicate whether each material, system or method listed is relevant to your study. If you are not sure if a list item applies to your research, read the appropriate section before selecting a response.

Materials & experimental systems

n/a	Involved in the study	
☐	☒	Antibodies
☒	☐	Eukaryotic cell lines
☒	☐	Palaeontology
☐	☒	Animals and other organisms
☒	☐	Human research participants
☒	☐	Clinical data

Methods

n/a	Involved in the study	
☐	☒	ChIP-seq
☒	☐	Flow cytometry
☒	☐	MRI-based neuroimaging

Antibodies

Antibodies used	RFP-TRAP, Chromotek rta-100
Validation	https://www.chromotek.com/products/nano-traps/rfp-trap/rfp-trapr-a/

Animals and other organisms

Policy information about studies involving animals; ARRIVE guidelines recommended for reporting animal research

Laboratory animals	*For laboratory animals, report species, strain, sex and age OR state that the study did not involve laboratory animals.*
Wild animals	*Provide details on animals observed in or captured in the field; report species, sex and age where possible. Describe how animals were caught and transported and what happened to captive animals after the study (if killed, explain why and describe method; if released, say where and when) OR state that the study did not involve wild animals.*
Field-collected samples	*For laboratory work with field-collected samples, describe all relevant parameters such as housing, maintenance, temperature, photoperiod and end-of-experiment protocol OR state that the study did not involve samples collected from the field.*
Ethics oversight	*Identify the organization(s) that approved or provided guidance on the study protocol, OR state that no ethical approval or guidance was required and explain why not.*

Note that full information on the approval of the study protocol must also be provided in the manuscript.

ChIP-seq

Data deposition

☒ Confirm that both raw and final processed data have been deposited in a public database such as GEO.

☒ Confirm that you have deposited or provided access to graph files (e.g. BED files) for the called peaks.

Data access links<br>*May remain private before publication.*	GEO:GSE122611

Files in database submission	GSM3474971 WUS-GR ChIP-Seq<br>GSM3474972 Col-0 ChIP-Seq<br>GSM3474973 WUS-GR Acetylation<br>GSM3474974 Col-0 Acetylation<br>GSM3474975 WUS-GR Methylation<br>GSM3474976 Col-0 Methylation
Genome browser session<br>(e.g. UCSC)	https://genome-euro.ucsc.edu/cgi-bin/hgTracks?<br>hgS_doOtherUser=submit&hgS_otherUserName=Olga_Ermakova&hgS_otherUserSessionName=hub_12832

Methodology

Replicates	One replicate from pooled seedlings for every condition
Sequencing depth	Read length 51 bp, single-end<br><br>GSM3474971 - WUS-GR ChIP-Seq, 25929753 reads, 16997410 reads mapped.<br>GSM3474972 - Col-0 ChIP-Seq, 22544513, 12278427 reads mapped.<br>GSM3474973 - WUS-GR Acetylation, 27905771 reads, 16976321 reads mapped.<br>GSM3474974 - Col-0 Acetylation, 13102823 reads, 6796500 reads mapped.<br>GSM3474975 - WUS-GR Methylation, 25175120 reads, 11182387 reads mapped.<br>GSM3474976 - Col-0 Methylation, 21708921, 9419455 reads mapped.
Antibodies	RFP-TRAP, Chromotek rta-100
Peak calling parameters	ChIP peak-calling was performed using Hiddendomains (v3.0) (Starmer, Joshua, and Terry Magnuson. "Detecting broad domains and narrow peaks in ChIP-seq data with hiddenDomains." BMC bioinformatics 17.1 (2016): 144) using following parameters: -q 15 (minimum MAPQ score), -b 100 (width of the bin) for WUS transcription factor, -b 500 for histone modification data, for the rest of parameters default settings were used.
Data quality	To ensure that our data were of high quality, we kept only called peaks with posterior probability higher than 0.9.
Software	ChIP-seq data were mapped to TAIR10 genome by BWA aligner (v0.7.17) on a local Galaxy instance (v17.09) (Afgan, Enis, et al. "The Galaxy platform for accessible, reproducible and collaborative biomedical analyses: 2018 update." Nucleic acids research 46.W1 (2018): W537-W544). Peak calling was performed using Hiddendomains (v3.0). Peaks were annotated to TAIR10 genes using PAVIS (Huang, Weichun, et al. "PAVIS: a tool for P eak A nnotation and Vis ualization." Bioinformatics 29.23 (2013): 3097-3099).
